# The moderating role of parental smoking on their children's attitudes toward smoking among a predominantly minority sample: a cross-sectional analysis

**DOI:** 10.1186/1747-597X-3-18

**Published:** 2008-07-14

**Authors:** Anna V Wilkinson, Sanjay Shete, Alexander V Prokhorov

**Affiliations:** 1 Department of Epidemiology, Unit 1340, The University of Texas M. D. Anderson Cancer Center, P.O. Box 301439, Houston, TX 77230, USA; 2 Department of Behavioral Science, Unit 1330, The University of Texas M. D. Anderson Cancer Center, P.O. Box 301439, Houston, TX 77230, USA

## Abstract

**Background:**

In general having a parent who smokes or smoked is a strong and consistent predictor of smoking initiation among their children while authoritative parenting style, open communication that demonstrates mutual respect between child and parent, and parental expectations not to smoke are protective. It has been hypothesized that parental smoking affects their children's smoking initiation through both imitation of the behavior and effects on attitudes toward smoking. The goals of the current analysis were to examine these two potential mechanisms.

**Methods:**

In 2003, 1,417 high school students in Houston, Texas, completed a cross-sectional survey as part of the evaluation of an interactive smoking prevention and cessation program delivered via CD-ROM. To assess the relationship between number of parents who currently smoke and children's smoking status, we completed an unconditional logistic regression. To determine whether the attitudes that children of smokers hold toward smoking are significantly more positive than the attitudes of children of non-smokers we examined whether the parents smoking status moderated the relationship between children's attitudes toward smoking and their ever smoking using unconditional logistic regressions.

**Results:**

Compared to participants whose parents did not currently smoke, participants who reported one or both parents currently smoke, had increased odds of ever smoking (OR = 1.31; 95% CI: 1.03–1.68; Wald χ^2 ^= 4.78 (df = 1) p = 0.03 and OR = 2.16; 95% CI: 1.51–3.10; Wald χ^2 ^= 17.80 (df = 1) p < 0.001, respectively). In addition, the relationship between attitudes and ever smoking was stronger among participants when at least one parent currently smokes (OR = 2.50; 95% CI: 1.96–3.19; Wald χ^2 ^= 54.71 (df = 1) p < 0.001) than among participants whose parents did not smoke (OR = 1.72; 95% CI: 1.40–2.12; Wald χ^2 ^= 26.45 (df = 1) p < 0.001).

**Conclusion:**

Children of smokers were more likely to smoke and reported more favorable attitudes toward smoking compared to children of non-smokers. One interpretation of our findings is that parental smoking not only directly influences behavior; it also moderates their children's attitudes towards smoking and thereby impacts their children's behavior. Our results demonstrate a continued need for primary prevention smoking interventions to be sensitive to the family context. They also underscore the importance of discussing parental smoking as a risk factor for smoking initiation, regardless of ethnicity, and of tailoring prevention messages to account for the influence that parental smoking status may have on the smoking attitudes and the associated normative beliefs.

## Introduction

Studies from the 1970s onwards have demonstrated that parental smoking and parental attitudes toward smoking are associated with smoking initiation among youth [e.g. [1-3]]. Flay et al. [4] reported that having a parent who smokes affects smoking initiation through imitation of the behavior and it also influences smoking attitudes, norms, and beliefs. Results from more recently published studies lend support to this claim. Having a parent who smokes or smoked is a strong and consistent predictor of smoking initiation among children [5-10], while authoritative parenting style [11,12], open communication that demonstrates mutual respect between child and parent [13,14], parental expectations not to smoke [15], and parental control [16] are protective.

To our knowledge, few, if any studies, have examined whether the strength of the reported association between attitudes toward smoking and ever smoking among children of smokers is significantly different from the strength of the association among children of non-smokers in a mostly minority sample. If children of smokers report significantly more positive attitudes toward smoking than children of non-smokers, this would suggest that having a parent who smokes modifies the relationship between attitudes and smoking, and provides evidence for an indirect effect of parental smoking on their children's behavior. In other words, parental smoking influences their children's attitudes toward smoking, which in turn increases the likelihood of the child smoking. This would suggest that interventions aimed at preventing adolescent smoking need to be sensitive to the family context and in particular underscores the need to discuss parental smoking with adolescents, especially with children of smokers.

Therefore our first goal was to examine the relationship between number of parents who currently smoked and children's ever smoking. We hypothesized that as the number of parents who currently smoke increases, so does the odds of their children's having ever smoked. Our second goal was to examine whether parental smoking modifies the relationship between children's attitudes toward smoking and children's ever smoking. We hypothesized that the relationship between attitudes and ever smoking is stronger when at least one parent is a current smoker.

## Methods

This study presents a secondary analysis of baseline cross-sectional data collected as part of the evaluation of A Smoking Prevention Interactive Experience (ASPIRE). ASPIRE is an interactive smoking prevention and cessation program delivered via CD-ROM that has been implemented and evaluated in eight high schools in Houston, Texas. For a detailed description of the study design, recruitment procedures, and instrumentation, see [17]. Participants were in grade ten when they completed the evaluation between September 2002 and January 2003. Active consent was obtained from all participants; parents were asked to return a signed informed consent and students who were 18 and over signed their own consent forms. All aspects of this study received approval from the Institutional Review Board at The University of Texas M.D. Anderson Cancer Center, the Committee for the Protection of Human Subjects at The University of Texas at Houston School of Public Health and from Houston Independent School District's Research Department.

### Participants

Participants included N = 1,417 or 88.6%, of the N = 1,599 high-school students in Houston who took part in the evaluation of ASPIRE. Of the 1,599 available for analysis 108 participants were missing data on the attitude measure and 74 were missing data on at least one of the parent variables.

### Dependent variable

The dependent variable in our study was ever smoking. We compared ever smokers (participants who had ever experimented with cigarettes) to never smokers, rather than simply comparing current experimenters to never smokers for two reasons. First, because we wanted to know if parental smoking influenced the odds of the child smoking, even just a puff of a cigarette, because 50% of children who even try a cigarette become smokers as adults [18]. And second, because smoking behavior at this age is quite volatile, it remains difficult to determine which child experimenter will become an adult smoker.

### Parental smoking effects

To examine the relationship between number of parents who currently smoked and children's ever smoking we classified parental smoking status as "neither smokes," "only one smokes," or "both smoke." To examine whether parental smoking modifies the relationship between children's attitudes toward smoking and children's ever smoking, we determined if there were differences in the strength of the relationship between children's attitudes toward smoking and ever smoking by parental smoking status. In other words we posed the question, are the attitudes that children of smokers hold toward smoking significantly more positive than the attitudes of children of non-smokers? We assessed participants' attitudes using the Temptations to Smoke Scale, which is composed of 10 items and is tailored to the participant's smoking status [19]. Current and former smokers answer identical versions of the scale, while never smokers answer a modified version [20]. Using only items that were common across both versions, we created a four-item scale; two items assess negative affect (e.g. "When things are not going my way and I'm frustrated"), and two items assess functional aspects of smoking (e.g. "When I want to get thinner"). The revised scale had very good internal reliability (Cronbach's alpha = 0.79) and was treated as both a continuous and binary variable. As a binary variable we compared children who reported no temptations to children who reported some temptations.

### Control variables

We controlled for the participants' gender, age (examined as a continuous variable), and ethnicity (coded as "Black," "Hispanic," "White," or "Other"). The "Hispanic" group served as the reference category. We also controlled for parents' current marital status (coded as "married" or "not married") and the highest level of educational attainment of either parent (coded as "college degree or more" or "less than college degree"). Parents who were "not married" and those with "less than college degree" served as the reference categories, respectively. "Not married" parents included the following categories: separated, divorced, single, and widowed.

### Statistical analyses

Bivariate associations between participant smoking status and participant gender, participant ethnicity, parents' highest level of educational attainment, parents' marital status, and parents' smoking status were assessed using chi-squares. For participant ethnicity we created a dummy variable for each ethnic group and conducted two by two chi-square analyses. We took the same approach with parents' educational attainment. Mean differences in age and children's smoking attitudes by participant smoking status were assessed using Student's *t*-tests.

To assess the relationship between number of parents who currently smoked (neither, only one, or both) and children's smoking status (ever vs. never), we completed an unconditional logistic regression, adjusting for the control variables.

To assess whether parental smoking modifies the relationship between children's attitudes toward smoking and children's ever smoking, we followed a methodology outlined by Baron and Kenny [21] to determine whether the attitudes that children of smokers hold toward smoking are significantly more positive than the attitudes of children of non-smokers. In other words we examined whether the parents smoking status moderated the participants' attitudes toward smoking on their ever smoking. Although moderation is best determined prospectively, the presence of a moderator effect based on cross-sectional data will lend support to our hypothesis, underscoring the need to confirm the effect using longitudinal data. For this analysis, we collapsed parental smoking status into a two-level variable (neither parent smokes vs. either parent smokes). Next, we created an interaction term between parental smoking status and children's smoking attitudes. All three variables were simultaneously entered into an unconditional logistic regression model, adjusting for the control variables. To further assess the interaction effect, we created a four-level categorical composite variable of parental smoking and attitudes. Categories included: a) neither parents smoke, child reports no temptations; b) neither parents smoke, child reports some temptations; c) a parent smokes, child reports no temptations; and d) a parent smokes, child reports some temptations. Next we completed an unconditional logistic regression, adjusting for the control variables again.

Having established moderation (presence of a significant interaction term), we stratified the sample on parental smoking status and completed two unconditional logistic regressions. Both models included children's smoking attitudes and adjusted for the control variables.

## Results

The participants included 569 males (40.1%) and 848 females (59.8%). The demographic characteristics of the participants and their parents by the participants' smoking status are presented in Table 1. The mean age of the participants was 15.66 years (SD = 0.90), and 587 (41.1%) reported ever smoking, of whom 17% reported smoking at least once every two weeks or more. Roughly 51% self-identified as Hispanic, 39% as black, and 6% as white; the remaining participants self-identified as Asian, American Indian, Alaska Native, Native Hawaiian, or Pacific Islander. The majority reported that their parents were married (52.3%) and that neither parent was a current smoker (55.9%); 32.3% reported that one parent currently smoked, and 11.8% reported that both parents currently smoked.

**Table 1:** Demographic characteristics of participants and their parents by participants' smoking status (N = 1,417)*

	**Participants' smoking status**			
		
	**Never (*n *= 830; 58.6%)**	**Ever (*n *= 587; 41.4%)**	**DF**	***p *value**
**Participant Characteristic**				
Gender				
Male	297 (52.2)	272 (47.8)		
Female	533 (62.9)	315 (37.1)	1	< 0.01
Age (years)				
Mean (SD)	15.60 (0.86)	15.74 (0.94)	1415	< 0.01
Ethnicity				
Black	375 (68.6)	172 (31.4)	1	< 0.01
Hispanic	376 (52.0)	347 (48.0)	1	< 0.01
Other	39 (62.9)	23 (37.1)	1	0.48
White	40 (47.1)	45 (52.9)	1	0.03
Temptations to smoke				
Mean (SD)	1.27 (0.65)	1.73 (0.90)	1415	< 0.01
**Parent Characteristic**				
Highest level of education of either parent				
Less than high school	136 (48.1)	147 (51.9)	1	< 0.01
Completed high school	188 (58.9)	131 (41.9)	1	0.88
Some college	157 (60.2)	104 (39.8)	1	0.57
Completed college	198 (63.3)	115 (36.7)	1	0.06
Marital status				
Married	463 (62.5)	278 (37.5)		
Not married	367 (54.8)	309 (45.2)	1	< 0.01
Smoking status				
Neither parent smokes	496 (63.6)	296 (36.4)		
Only one parent smokes	260 (56.8)	198 (43.2)		
Both parents smoke	74 (44.3)	93 (55.7)	2	< 0.01

*Data in table are numbers of participants or parents (percentages) unless otherwise specified; percentages are row percentages.

SD = Standard Deviation; DF = Degrees of Freedom.

P-values for age and temptations to smoke are based on Student's t-test, all other p-values are based on chi-square tests of association.

Results from the multivariable unconditional logistic regression examining the influence of the number of parents who currently smoked on children's ever smoking are presented in Table 2. Compared to their peers who reported that neither parent currently smoked, participants who reported that one parent currently smoked were 1.31 times (95% confidence interval [CI] = 1.03–1.68; Wald χ^2 ^= 4.78 (df = 1) p = 0.03) as likely to have ever smoked, and participants who reported that both parents currently smoked were 2.16 times (95% CI = 1.51–3.10; Wald χ^2 ^= 17.80 (df = 1) p < 0.001) as likely to have ever smoked. In addition, the overall p-value for number of parents who smoke was significant (p for trend < 0.001). Ever smoking also was associated with being male and older, living with parents' who highest level of education was less than a high school degree, while being black and living with parents who are married were protective.

**Table 2:** Unconditional logistic regression examining the influence of number of parents who smoke on children's ever smoking (N = 1,417)

**Characteristic**	**OR**	**95% CI**		***p *value**
Male	1.63	1.30	2.05	< 0.01
Age in years	1.14	1.01	1.29	0.04
Black vs. Hispanic	0.44	0.34	0.57	0.00
Other vs. Hispanic	0.68	0.39	1.18	0.17
White vs. Hispanic	1.05	0.65	1.69	0.86
Some HS vs. completed college	1.65	1.21	2.26	0.01
Completed HS vs. completed college	1.21	0.90	1.63	0.21
Some college vs. completed college	1.33	0.96	1.84	0.08
Parents are married	0.59	0.47	0.75	< 0.01
Only one parent smokes	1.31	1.03	1.68	0.03
Both parents smoke	2.16	1.51	3.10	< 0.01

OR, odds ratio; CI, confidence interval; ORs are adjusted for all other variables presented in the table. P-values are based on Wald chi-square with 1 df.

To determine if the attitudes that children of smokers hold toward smoking are significantly more positive than the attitudes of children of non-smokers (i.e. whether parental smoking status potentially moderates participants' attitudes toward smoking), we first examined the interaction term between parental smoking status and children's smoking attitudes (Baron and Kenny, 1986). After controlling for the participants' gender, age, and ethnicity, the parents' educational attainment and marital status, and the two main effects (children's attitudes and parental smoking status) the interaction term was significant (OR = 1.27; 95% CI = 1.07–1.52; Wald χ^2 ^= 7.41 (df = 1) p < 0.01; data not shown). In other words, having at least one parent who currently smoked potentially does moderate the influence of children's smoking attitudes on ever smoking.

**Figure 1:**
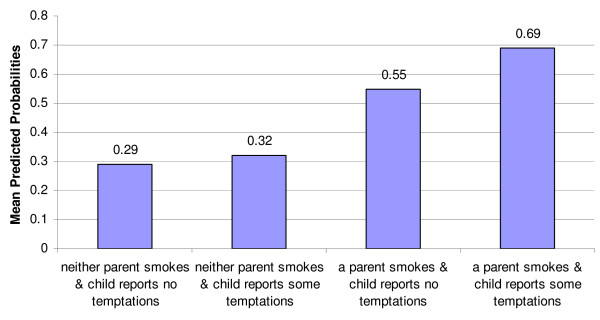
Mean Predicted Probabilities of Ever Smoking by Parental Smoking Status and Temptations to Smoke.

Results from the multivariable unconditional logistic regression examining the four-level categorical variable are presented in table 3. Compared to children whose parents do not smoke and who reported no temptations to smoke, their peers who reported some temptations were no more of less likely to be ever smokers; however children who live with at least one parent who smokes were more likely to be ever smokers. Among children who reported no temptations, the odds of ever smoking were 2.89 (95% CI = 2.09–3.97; Wald χ^2 ^= 41.49 (df = 1) p < 0.01), but among the children who reported temptations, the odds of ever smoking were 5.26 (95% CI = 3.74–7.40; Wald χ^2 ^= 91.42 (df = 1) p < 0.01). Please see figure 1 for the predicted probabilities of smoking based on the four-level categorical variable.

**Table 3:** Unconditional logistic regression examining the interaction between having a parent who smokes and child's attitudes toward smoking on children's ever smoking (N = 1,417)

**Characteristic**	**OR**	**95% CI**		***p *value**
Neither parent smokes, child reports no temptations	1.00			
Neither parent smokes, child reports some temptations	1.16	0.864	1.56	0.32
A parent smokes, child reports no temptations	2.89	2.09	3.97	< 0.01
A parent smokes, child reports some temptations	5.26	3.74	7.40	< 0.01

OR, odds ratio; CI, confidence interval; ORs are adjusted for all other variables presented in the table. P-values are based on Wald chi-square with 1 df.

Control variables include child's age, gender, ethnicity and parents' educational attainment and marital status.

Next, we stratified the sample by parental smoking status and conducted multivariable logistic regression analyses to investigate the relationship between children's smoking attitudes and ever smoking (Table 4). Among participants whose parents were current nonsmokers, children's smoking attitudes were associated with a 1.72 times (95% CI = 1.40–2.12; Wald χ^2 ^= 26.45 (df = 1) p < 0.001) increased risk of being an ever smoker, whereas among participants who had at least one parent who currently smoked, children's smoking attitudes were associated with a 2.50 times (95% CI = 1.96–3.19; Wald χ^2 ^= 54.71 (df = 1) p < 0.001) increased risk of being an ever smoker. Among participants whose parents were current nonsmokers, being male and older, as well having parents who have not completed high school were associated with ever smoking, while being black and having married parents were protective. Among participants with at least one parent who currently smoked, being male and having parents who have not completed high school were associated with ever smoking, while being black and parents' being married were protective.

**Table 4:** Unconditional logistic regressions examining the influence of children's attitudes on their ever smoking, by parental smoking (N = 1,417)

	**Neither parent smokes (*n *= 792)**				**At least one parent smokes (*n *= 625)**			
	
**Characteristic**	**OR**	**95% CI**		***p *value**	**OR**	**95% CI**		***p *value**
Male	1.64	1.20	2.25	< 0.01	1.72	1.20	2.47	< 0.01
Age in years	1.21	1.02	1.42	0.03	0.98	0.80	1.20	0.82
Black vs. Hispanic	0.42	0.29	0.62	0.00	0.57	0.38	0.87	< 0.01
Other vs. Hispanic	0.66	0.31	1.39	0.66	0.81	0.33	2.00	0.65
White vs. Hispanic	1.00	0.49	2.03	0.99	1.23	0.61	2.50	0.57
Some HS vs. completed college	1.53	1.00	2.35	0.05	2.22	1.34	3.68	< 0.01
Completed HS vs. completed college	1.33	0.88	2.00	0.18	1.38	0.87	2.19	0.18
Some college vs. completed college	1.45	0.92	2.29	0.14	1.45	0.88	2.38	0.15
Parents are married	0.62	0.45	0.86	< 0.01	0.69	0.48	1.00	0.05
Temptations to smoke	1.72	1.40	2.12	0.00	2.50	1.96	3.19	< 0.01

OR, odds ratio; CI, confidence interval; ORs are adjusted for all other variables presented in the table. P-values are based on Wald chi-square with 1 df.

## Discussion

In this study, we explored two mechanisms through which parental smoking may influence their children's smoking behavior and found evidence for both mechanisms. Consistent with previous research [22-24], we found that the odds for smoking increased with the number of parents who currently smoked. Compared to participants whose parents did not currently smoke, participants who reported that one parent currently smoked had a 1.3 times increased risk for ever smoking, and those who reported that both parents currently smoked had a 2.2 times increased risk. The p for trend was significant, suggesting a dose-response relationship. Growing up in a family in which one or both parents smoke not only provides children with the opportunity to imitate the behavior, living with two parents who smoke as compared to one or none, increases access to cigarettes [25,26]. In addition, for many children, living in a household where one or both parents smoke means that they have been second-hand smokers, are used to breathing smoke, and therefore may not have a physically aversive reaction to tobacco when they first try.

We also found that the association between children's smoking attitudes and ever smoking was stronger when at least one parent currently smoked. Among participants whose parents did not currently smoke, children's smoking attitudes were associated with a 1.7 times increased risk for ever smoking, whereas among participants who reported that at least one parent currently smoked, children's smoking attitudes were associated with a 2.5 times increased risk. Our results suggest that parental smoking influences children's attitudes toward smoking, which in turn affect the likelihood of the child smoking. To the best of our knowledge, although one previous study examined whether parental smoking mediates (is an intermediate step in the casual pathway) the relationship between children's attitudes toward smoking and smoking behavior [4] and another examined the relationship between children's implicit and explicit attitudes toward smoking and parental smoking [27], no studies have examined if parental smoking status moderates (modifies) the relationship between children's attitudes toward smoking and their ever smoking. However, because moderation is best determined prospectively, our results lend support to the hypothesis and need to be confirmed using longitudinal data.

Our results differ from those of the Chassin et al. [27] study, which found that neither the children's implicit nor their explicit attitudes toward smoking were associated with parental smoking status. Our results, however, are consistent with, and extend the earlier findings of Flay et al. [4], who noted that parental smoking was associated with changes in adolescent's explicit attitudes. Not only did we observe an overall association between parental smoking and children's explicit attitudes, we found that the relationship between children's attitudes toward smoking and their ever smoking was weaker among children of parents who did not currently smoke compared to children whose parents currently smoke. Again, because moderation is best determined prospectively, our results lend support to the hypothesis that parental attitudes moderate this relationship.

It is of concern that the relationship between attitudes toward smoking and ever smoking is stronger when at least one parent smokes because positive attitudes toward smoking among youth are associated with increased susceptibility to smoking [28], and youth who have experimented with cigarettes or who currently smoke [27,29,30] hold significantly more positive attitudes toward smoking than do their nonsmoking peers. Moreover, Chassin et al. [27] found that mothers who smoke report significantly more positive implicit and explicit attitudes toward smoking than mothers who have quit or never smoked, underscoring the possibility that positive attitudes toward smoking are learned and sanctioned at home.

The participants in the Chassin et al. [27] were predominantly non-Hispanic white, as were the majority of the participants in Ridner's study [30]. However, the participants in the Chalela study were all Latino, while the sample in the Flay et al. study [4] was multi-ethnic. Although the goals of these four studies and ours were different and all used different analytic approaches, all studies examined the relationship between attitudes toward smoking and behavior and all reported consistent results: positive attitudes were associated with ever smoking. Taken as a whole, this suggests that consistent with the conclusions drawn from other studies based on multi-ethnic samples [9,10], predictors of smoking behavior may be universal.

While our study focused on the family context, other factors may impact the relationship between children's attitudes towards smoking and their ever-smoking. For example, many studies have documented the role that peer influence [31,32] and perceptions of peer norms [33] play on smoking initiation. While outside the scope of the current study, examining peer influence, both separate from and in conjunction with parental influence, would refine our understanding of the relationship between attitudes and smoking.

This study has some limitations. First, the analysis is based on self-reported cross-sectional survey, limiting our ability to draw causal conclusions and test for moderation. Second, while most contemporary approaches to assessing family structure tend to compare differences between single and two parent families without regard to marital status, the data collected in this study did not permit such a distinction. The response categories probing parental marital status included married, separated, divorced, single, and widowed. Therefore we examined the influence of reporting married parents, which may serve as a proxy for two parent households, compared to reporting that parents are separated, divorced, single, or widowed. In future research we intend to fully examine the relationship between household structure, living arrangements and number of parents who smoke. Third, we do not know how long the participants were exposed to parental smoking, which limits our ability to determine if there is a threshold of exposure required to influence children. Fourth, we did not ask the ever smokers where they obtained the cigarettes they smoked. Therefore we cannot determine if current parental smoking directly increases access, and we cannot control for its potential influence in the analysis. Fifth, active consent was required of all students to participate in this study; more girls than boys returned their consent form resulting in the differential participation rates. Finally, we did not examine the influence of exposure to a parent who quit smoking while the participant was growing up, which has been shown to increase the likelihood of smoking [24]. However, the net result from the lack of information about past parental smoking would have underestimated the effect sizes observed in our study (biased the results toward the null), suggesting our findings may have been more pronounced had we adjusted for past parental smoking.

## Conclusion

In conclusion, our study found evidence for two potential mechanisms through which one current parental smoking may influence children's smoking behavior. Parental smoking influences children's smoking independent of impacting child attitudes as well as influencing their children's attitudes toward smoking, which in turn may increase the likelihood of the child smoking. In addition to the mechanisms identified in our analysis, others have demonstrated that current parental smoking is associated with increased access to cigarettes [25], while living in a two parent household is protective [31,33]. Our results refine our understanding of how the family context contributes to smoking initiation, thereby demonstrating a continued need for primary prevention smoking interventions to be sensitive to the family context. Specifically, our results underscore the importance of discussing parental smoking as a risk factor for smoking initiation, regardless of ethnicity, and of tailoring prevention messages to account for the influence that parental smoking status may have on the smoking attitudes and the associated normative beliefs held by children of smokers.

## Authors' contributions

AVW conceived the current analysis, completed the analysis, and led the writing. SS oversaw the analysis and provided critical revisions. AVP conceived the original study, interpreted the results, and provided critical revisions.
